# Mr. Skinner on Our General Index

**Published:** 1810-10

**Authors:** I. S. Skinner


### *To the Editors of the Medical and Physical Journal.*

GENTLEMEN,

I HAVE been a constant reader and admirer of your periodical work, ever since its commencement, and was happy to find by your communication a few months back, that you had not laid aside your long promised general index, at first proposed to include the 20th volume, but by the late account, it is to appear as soon as possible after the completion of the 24th volume*, when I should suppose you mean to commence a new series; and if you do, I have just taken the liberty to suggest a few hints for your consideration. Might it not be a very desirable thing to give us a new history of physic? Mention the different opinions and practices of such physicians, and others who have shone in the profession; also an account of such of their works, as have rendered them eminent; and likewise, if you were to point out as you proceed, such of their publications, as are worth attending to (for I have no doubt whatever, but there is a great deal of useful knowledge to be collected from the authors of the 16th, 17th, and 18th centuries), at this juncture; you would do an essential service to practitioners in general, and to the students in particular, who have scarce any directions to guide them by at present. We also stand in need of a complete catalogue of medical works, but whether this, or

* It will appear immediately after the publication of the thirtieth volume, or first series of this work; after which, a new series will be commenced.

my my other hints may be deemed worthy of your attention or time, I cannot tell, but as they occurred to me, as likely to promote the sale and circulation of your very valuable work, I have taken the liberty to mention them, and remain your constant reader, I. S. SKINNER.

*Sept.* 19, 1810.

